# Severe protein C deficiency in a newborn caused by a homozygous pathogenic variant in the *PROC* gene: a case report

**DOI:** 10.1186/s12887-021-02923-6

**Published:** 2021-10-16

**Authors:** Uisook Song, Young Hye Ryu, Kiteak Hong, So-Yeon Shim, Seongyeol Park, Jeong Seok Lee, Young Seok Ju, Seung Han Shin, Soyoung Lee

**Affiliations:** 1 Department of Pediatrics, Seoul National University Hospital, Seoul National University College of Medicine, 101 Daehak-ro, Jongno-gu, Seoul, 03080 Republic of Korea; 2 Department of Pediatrics, College of Medicine, Ewha Womans University, Seoul, Republic of Korea; 3 GENOME INSIGHT Inc., Daejeon, Republic of Korea; 4 Graduate School of Medical Science and Engineering, Korea Advanced Institute of Science and Technology, Daejeon, Republic of Korea

**Keywords:** Severe protein C deficiency, Coagulopathy, *PROC* gene

## Abstract

**Background:**

Severe protein C deficiency is a rare and inherited cause of thrombophilia in neonates. Protein C acts as an anticoagulant, and its deficiency results in vascular thrombosis. Herein, we report a case of protein C deficiency with a homozygous pathogenic variant in a term neonate, with good outcomes after proper treatment.

**Case presentation:**

A four-day-old male newborn was transferred to the Seoul National University Hospital on account of dark red to black skin lesions. He was born full-term with an average birth weight without perinatal problems. There were no abnormal findings in the prenatal tests, including intrauterine sonography. The first skin lesion was observed on his right toes and rapidly progressed to proximal areas, such as the lower legs, left arm, and buttock. Under the impression of thromboembolism or vasculitis, we performed a coagulopathy workup, which revealed a high D-dimer level of 23.05 μg/ml. A skin biopsy showed fibrin clots in most capillaries, and his protein C activity level was below 10%, from which we diagnosed protein C deficiency. On postnatal day 6, he experienced an apnea event with desaturation and an abnormal right pupillary light reflex. Brain computed tomography showed multifocal patchy intracranial hemorrhage and intraventricular hemorrhage with an old ischemic lesion. Ophthalmic examination revealed bilateral retinal traction detachments with retinal folds. Protein C concentrate replacement therapy was added to previous treatments including steroids, prostaglandin E1, and anticoagulation. After replacement therapy, there were no new skin lesions, and the previous lesions recovered with scarring. Although there were no new brain hemorrhagic infarctions, there was ongoing ischemic tissue loss, which required further rehabilitation. Ophthalmic surgical interventions were performed to treat the bilateral retinal traction detachments with retinal folds. Molecular analysis revealed a homozygous pathogenic variant in the *PROC* gene.

**Conclusion:**

Severe protein C deficiency can manifest as a fatal coagulopathy in any organ. Early diagnosis and proper treatment, including protein C concentrate replacement, may improve outcomes without serious sequelae.

## Background

Protein C is a vitamin K-dependent plasma glycoprotein zymogen that is synthesized in hepatocytes and activated by the thrombin-thrombomodulin complex [1]. Protein C deficiency causes macro- and microvascular thrombosis [2]. It could be identified when the protein C concentration or activity is below normal (lower limit of normal plasma concentration of protein C in healthy term infant, 25 IU/dL) [3]. Protein C deficiency is a rare genetic disorder caused by alterations in the *PROC* gene located on chromosome 2q14.3 [2]. The incidence of protein C deficiency is 1 in 40,000 to 250,000 individuals [4]. In particular, homozygous protein C deficiency is rare, with an incidence of 1 in 500,000 to 750,000 individuals [3, 5]. Both autosomal dominant and autosomal recessive inheritance can lead to disease development. Patients with a heterozygous pathogenic variant experience relatively late-onset recurrent venous thrombosis and decreased plasma protein C levels and protein C activity levels [3]. They often present with venous thromboembolism but may also be asymptomatic. On the other hand, patients with homozygous or compound heterozygous protein C deficiency have more severe symptoms that typically cause purpura fulminans and severe disseminated intravascular coagulation (DIC) with severe venous thromboembolism in the neonatal period. These patients have very low protein C activity [1, 3].

Herein, we describe the case of a newborn with protein C deficiency caused by a homozygous pathogenic variant in the *PROC* gene, who was treated with protein C concentrate.

## Case presentation

**Fig. 1:**
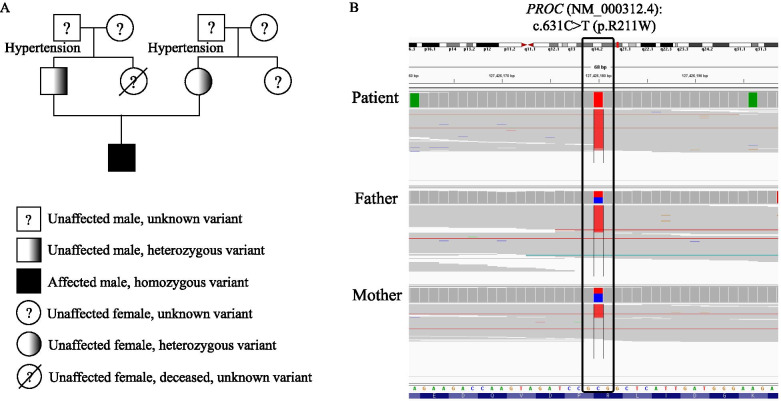
Family pedigree and genetic analysis of the patient and his parents. (**A**) Family pedigree of patient. (**B**) Genetic analysis of the patient and his parents. The Intergrative Genomic Viewer (IGV) demonstrates homo- and heterozygous variants of the *PROC* gene (NM_000312.4). The 631th reference base of coding sequence in *PROC* gene is C (blue color), but the patient has only T (red color) and his parents have both C and T

**Fig. 2:**
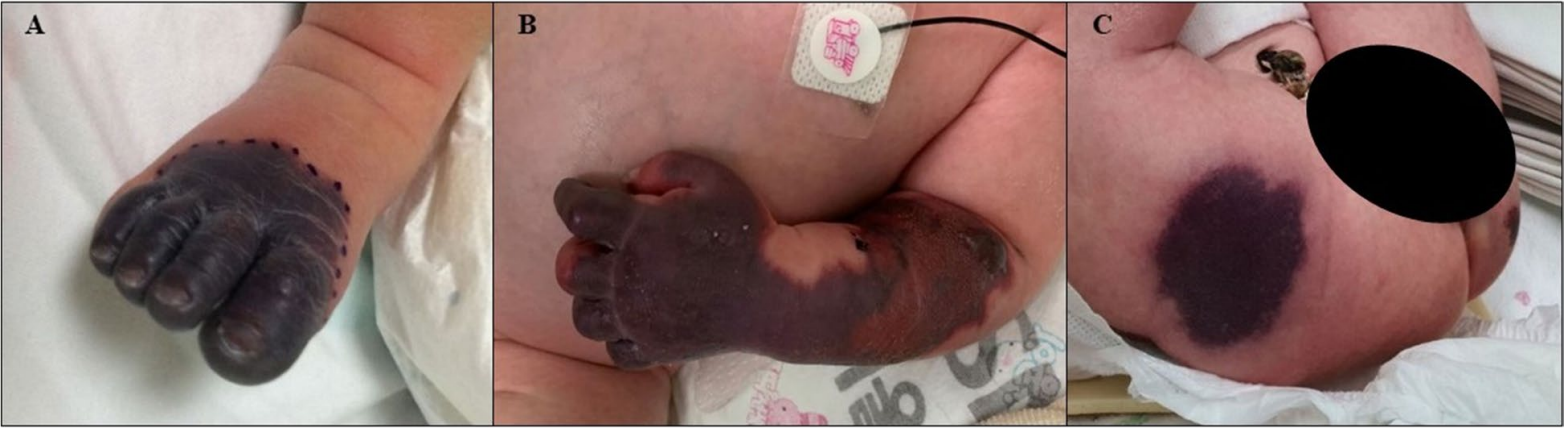
Skin changes of newborn with purpura fulminans

A four-day-old male newborn was transferred to the Seoul National University Hospital on account of progressive necrotic skin lesions. He was delivered via cesarean section due to prolonged labor at a gestational age of 39 weeks + 2 days with a birth weight of 3560 g. The patient was an only child with no remarkable prenatal or perinatal problems, including intrauterine sonography findings. Both parents denied a family history of hematologic or autoimmune diseases (Fig. 1A). He was initially treated with antibiotics under the impression of neonatal sepsis due to necrotic skin change at an outside hospital; however, his skin lesions worsened. The skin lesions started at the toes and rapidly progressed to the lower legs, buttocks, left hand, and left upper arm. The lesions were initially dark red and then changed to black (Fig. 2).

On the sixth postnatal day, he abruptly experienced several episodes of apnea accompanied by desaturation, from which he soon recovered. Although he did not show motor deficits, his right pupil was unresponsive to light, and this was followed by clonic movements in both arms for a few seconds.

**Fig. 3:**
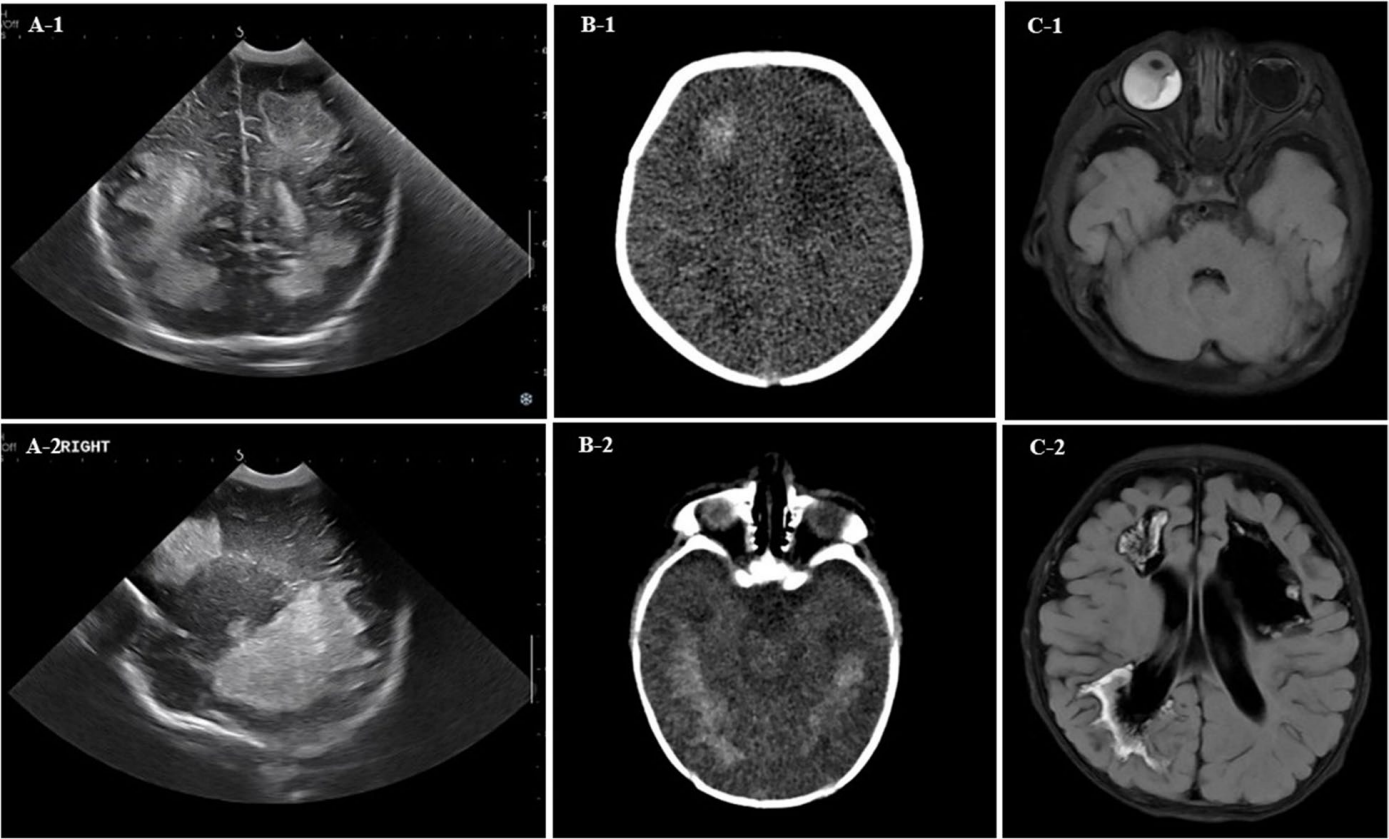
Brain images of the patient. (**A**) Brain sonography of neonate on postnatal day 6 showing recent onset hemorrhagic infarction and liquefied infarction from possible in-utero insult. (**B**) Brain computer tomography (CT) images of neonate on postnatal day 6 showing intracranial hemorrhage, old ischemic lesions, and suspicious intraventricular hemorrhage. (**C**) Brain MRI (T2 FLAIR image) obtained at 1 month of age, showing cystic cerebromalatic changes in the bilateral periventricular white matter, diffusion restrictions along the margin of the previously infarcted area, suggesting ongoing loss of ischemic tissue, and right retinal detachment with vitreous hemorrhage

Brain sonography and computed tomography images showed multifocal patchy intracranial hemorrhage in the right cerebral hemisphere and left temporo-occipital area, and intraventricular hemorrhage without ventricular dilation. Moreover, an old ischemic lesion, which was thought to have occurred in the uterus, was observed on the left periventricular white matter (Fig. 3A, B). Brain magnetic resonance imaging (MRI) and brain sonography images did not reveal venous thromboembolism (Fig. 3C). Venous sonography images showed thrombotic occlusion of the left cephalic vein, but not of the internal organs and veins, such as the kidney, liver, spleen, pancreas, and portal vein. Electroencephalography showed intermittent, slightly suppressed background activity and occasional spike discharges, which suggested diffuse cerebral dysfunction and focal seizures.

The complete blood count, liver function test, and renal function test results were unremarkable except for hyperbilirubinemia, which was thought to be due to physiologic jaundice. The levels of inflammatory markers were not elevated, and microorganisms were not isolated in the blood, urine, or cerebrospinal fluid. The prothrombin time international normalized ratio (PT-INR), activated partial thromboplastin time (aPTT), and D-dimer levels were 0.99 s, 33 s, and 23.05 μg/ml, respectively (Table 1). The protein C activity level was remarkably decreased below 10% (normal range: 74–152%); however, the protein S concentration was within the normal range. A skin biopsy of the left calf skin lesion showed fibrin clots in most capillaries, suggesting DIC or deep vein thrombosis.

**Table 1:** Results of complete blood count, coagulation work up, and D-dimer levels

	Patient	Reference range
Complete blood count		
White blood cell (/μL)	17,380	6000-17,500
Hemoglobin (g/dL)	14.3	10.5–13.5
Hematocrit (%)	40.4	33–39
Platelets (10^3^/μL)	232	130–400
Coagulation		
PT-INR (INR)	0.99	0.8–1.2
PT (%)	101	80–120
PT (sec)	11.8	10.6–12.9
aPTT (sec)	33.0	25.1–36.2
Fibrinogen (mg/dL)	153	192–411
Antithrombin (%)	79	81–126
D-dimer (μg/mL)	23.05	0.04–0.49
Protein C activity (%)	< 10	74–152
Protein C Ag (%)	20.04	72–160
Protein S (%)	86	73–150

Molecular analysis was performed targeting inherited diseases presenting as early-onset vasculitis or coagulopathy. The results revealed a homozygous pathogenic variant c.631C > T (p. Arg211Trp) in the *PROC* gene, which was inherited from both his unaffected parents (Fig. 1B). This variant is seldom present in the population database (rs121918143, ExAC 0.02%) and has already been observed to be a compound heterozygous variant in individuals affected by purpura fulminans and severe protein C deficiency [6]. Both parents showed no clinical signs of heterozygous mutation; the father and mother showed protein C activity levels of 69 and 57%, respectively (normal range, 74–152%).

The patient was initially treated with intravenous prostaglandin E1, high-dose steroids, intravenous immunoglobulin with antibiotics, and nitroglycerin ointment to prevent skin necrosis prior to the diagnosis of severe protein C deficiency. Antiepileptic drugs and mannitol were added to prevent further seizures and decrease the intracranial pressure, respectively. Under the diagnosis of protein C deficiency, fresh frozen plasma (FFP) transfusions (10 ml/kg/day) were started.

As new skin lesions were found on the left arm and both buttocks on postnatal day 7, low-molecular-weight heparin (LMWH) treatment was started. Protein C concentrate replacement therapy was initiated on postnatal day 11. The first dose of protein C concentrate was 100 IU/Kg, followed by 50 IU/Kg every 8 h. The doses of protein C concentrate were adjusted according to protein C activity level and clinical symptoms. The target level of protein C activity was 50% and the dose of protein C concentrate was increased if the existing necrotic skin lesions were aggravated or a new lesion was developed. As a result, protein C concentrates were given up to 80 IU/kg every 8 h. The immediate level of protein C activity after 1st loading of protein C concentrate was 47%, and the final trough level was 55% on postnatal days 12 and 17, respectively. The level of D-dimer was decreased to 5.13 μg/mL after protein C concentrate replacement but was not normalized. From postnatal day 17, anticoagulation therapy was changed to warfarin instead of protein C replacement and heparinization therapy. The target PT-INR was 2.0 to 3.0 [7].

**Fig. 4:**
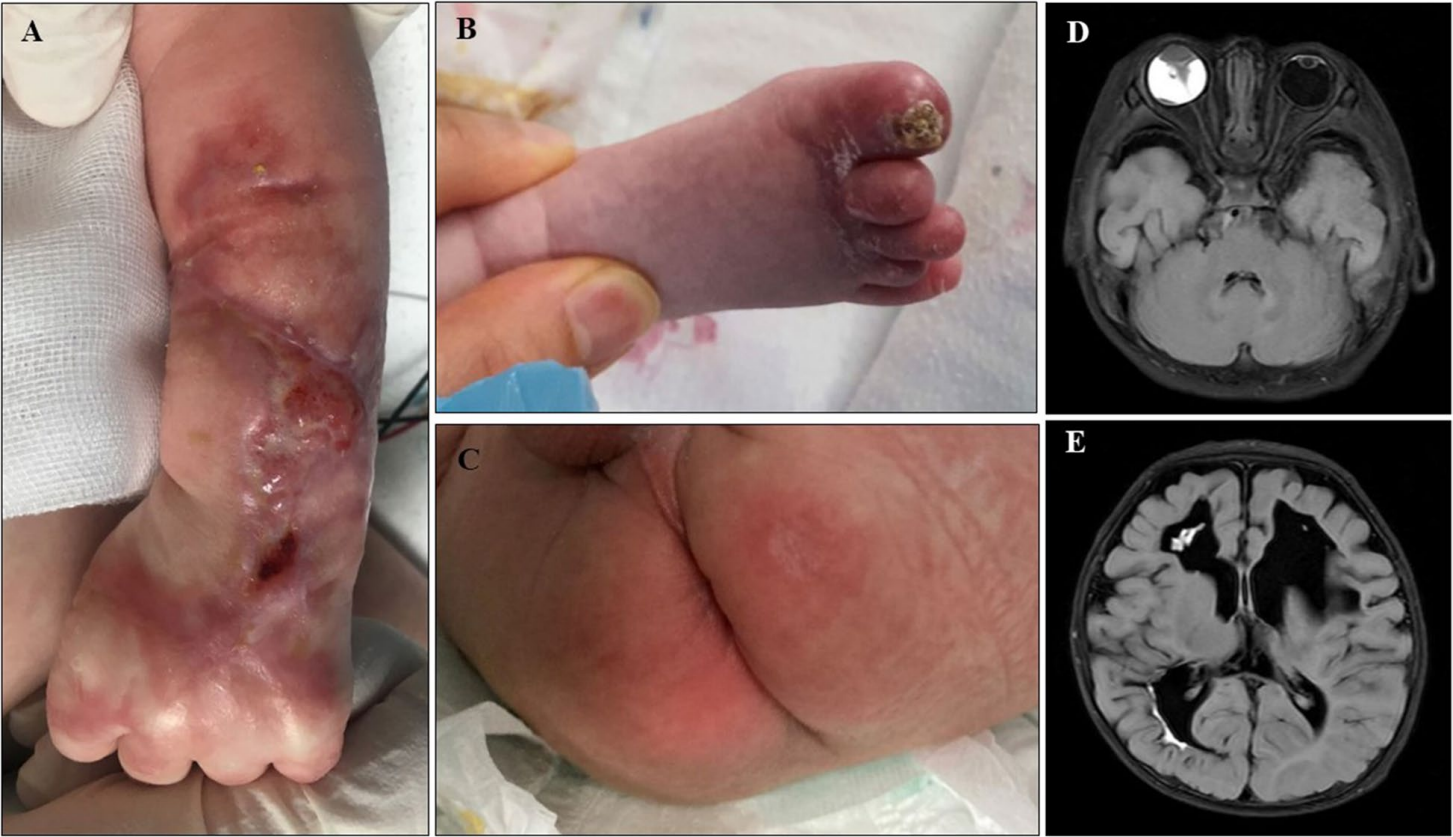
(**A**–**C**) Healed lesions at 2 months of age after replacement therapy with protein C concentrate. (**D**–**E**) Brain MRI (T2 FLAIR image) obtained at 2 months of age showing remaining diffusion restrictions along previously infarcted areas, suggesting ongoing loss of ischemic tissue

After 2 months of treatment, the patient’s skin lesions healed without any surgical treatment but remained scarred with shortened toes (Fig. 4A–C). There were no more clinical seizures, and follow-up brain sonography and brain MRI did not show any new hemorrhagic brain infarctions; however, there was ongoing loss of ischemic tissue (Fig. 4D, E). Ophthalmic surgical interventions were performed to treat bilateral retinal traction detachments with retinal folds. Finally, the patient was discharged on postnatal day 87 with warfarin and other medications.

## Discussion and conclusions

Homozygous or compound heterozygous protein C deficiency is a rare disorder characterized by very low protein C activity and may be life-threatening, causing purpura fulminans in the neonatal period [8]. In this paper, we reported a case of a patient with a homozygous pathogenic variant of the *PROC* gene (c.631C > T, p.Arg211Trp). Each allele was inherited from his parents, who were unaffected. This patient experienced early-onset neonatal purpura fulminans, intracranial hemorrhage, and bilateral retinal detachments. The patient likely developed intracranial hemorrhage and bilateral retinal detachments during the prenatal period. These grave symptoms were well treated with protein C concentrates, FFP replacements, LMWH, warfarin, and ophthalmic surgery, leading to rehabilitation and discharge from the hospital.

There have been previous reports of protein C deficiency, presenting with neonatal purpura fulminans; intracranial hemorrhage or infarctions; and ophthalmologic complications including non-reactive pupils; leukocoria; vitreous, retinal, and subretinal hemorrhage; retinal arterial and venous occlusion; kidney infarction; and rarely, colonic perforation [1, 9, 10]. In most cases, treatments such as FFP replacement, LMWH, warfarin, and protein C concentrates were used effectively. Among these treatments, protein C concentrates are the most effective, as shown in this patient; however, they are very expensive and not always available in most countries. Some patients underwent surgical interventions such as ventriculoperitoneal shunt placement due to subarachnoid and intraventricular hemorrhage [11]. Liver transplantation may be considered a curative therapy for severe congenital protein C deficiency [1]. Early diagnosis and progressive treatment will lead to minimally invasive treatments and further reduce sequelae. As shown in the present case, thrombotic events could occur during the prenatal period. However, it was difficult to detect thrombosis or hemorrhage via prenatal examinations.

At least 200 pathogenic variants in the *PROC* gene are known to cause protein C deficiency, and most variants are heterozygous [12]. While most homozygous or compound heterozygous protein C deficient patients had poor outcomes, there is a case report of patients with homozygous protein C deficiency presenting with a milder course with low, measurable protein C levels [9]. This suggests that other factors may influence gene penetrance [13]. This variant was observed to not only be homozygous or compound heterozygous in individuals with severe protein C deficiency, but also to be heterozygous in individuals with mild thrombosis or segregated with disease [6, 8, 14–17]. It is observed more frequently in Korean patients as well as in the normal population [16].

Congenital protein C deficiency requires long-term treatment with regular check-ups. In a case report describing the rehabilitation of a patient with brain damage due to protein C deficiency, it was recommended that purpura fulminans caused by insufficient anticoagulation should be differentiated from hematoma caused by excessive anticoagulation therapy [18]. Further studies are needed to establish appropriate long-term treatment guidelines with LMWH or warfarin for a better prognosis.

In conclusion, we genetically diagnosed and treated a patient with severe protein C deficiency with a homozygous pathogenic variant presenting with neonatal purpura fulminans, intracranial hemorrhage and infarct, and bilateral retinal detachments. We hope that our report will further assist other clinicians in diagnosing and treating this rare disease.

## Data Availability

All data generated or analyzed during this study are included in this published article.

## Abbreviations

aPTT: Activated partial thromboplastin time
CT: Computed tomography
FFP: Fresh frozen plasma
IV: Intravenous
MRI: Magnetic resonance imaging
*PROC* gene: protein C gene
PT-INR: Prothrombin time-international normalized ratio
